# Selecting Hydrogel Films Composed of Carboxymethyl Cellulose and Microcrystalline Cellulose From OPEFB With Citric Acid as a Green Crosslinker for Fruit Wrapping

**DOI:** 10.1155/ijbm/2806425

**Published:** 2026-02-17

**Authors:** Susi Susi, Makhmudun Ainuri, Wagiman Wagiman, Mohammad Affan Fajar Falah, Hisyam Musthafa Al Hakim

**Affiliations:** ^1^ Department of Agroindustrial Technology, Faculty of Agriculture, Lambung Mangkurat University, Jl A Yani Km 36, Banjarbaru, 70714, South Kalimantan, Indonesia, unlam.ac.id; ^2^ Department of Agroindustrial Technology, Faculty of Agricultural Technology, Gadjah Mada University, Jl Flora No 1 Bulaksumur, Yogyakarta, 55281, Indonesia, ugm.ac.id

## Abstract

Cellulose‐based hydrogel films are promising alternatives to plastic for wrapping. Hydrogel films based on carboxymethyl cellulose (CMC) and microcrystalline cellulose (MCC) must exhibit high mechanical strength and be capable of absorbing moisture. Crosslinking increases the absorption capacity and mechanical strength of hydrogel films. As a filler, MCC can synergize with citric acid, which acts as an environmentally friendly crosslinker to enhance mechanical strength. This research aimed to develop a suitable CMC/MCC formulation and citric acid as a crosslinker, resulting in a hydrogel with high water absorption and mechanical strength. CMC/MCC formulations were combined with citric acid concentrations of 5%, 7.5%, and 10%. The results showed that the MCC and citric acid will affect absorption and mechanical strength. The addition of MCC up to 50% tends to produce a brittle hydrogel film, and this phenomenon is also correlated with increased citric acid. A 90:10 CMC/MCC formulation with 5% citric acid (w/v) resulted in a water uptake of 222.72 ± 9.32 at pH 7.0. In contrast, the 80:20 CMC/MCC formulation resulted in a water uptake of 603.02 ± 26.98. They both showed higher rehydration of the dry gel than the others. FTIR confirmed the sharpening of the peak wave number at 1705 cm^−1^, which is identical to the protonated carbonyl group and correlates with the water absorption capacity. The morphology of hydrogel films containing a CMC/MCC ratio of 90:10 exhibits a smoother surface than that of those with a CMC/MCC ratio of 80:20, which feature bubbles on the surface cracks of the hydrogel film due to the presence of more water absorption channels. Hydrogel films with a CMC/MCC ratio of 90:10 and 5% citric acid (w/v) can be developed for wrapping by modifying their hygroscopic properties. In contrast, hydrogel films with an 80:20 ratio and 5% citric acid are suitable for use as absorbents.

## 1. Introduction

Plastic wrapping remains a highly preferred material for food packaging and other single‐use applications due to its low cost, durability, and convenience. However, its non‐biodegradability and contribution to environmental degradation have highlighted the urgent need for sustainable and eco‐friendly alternatives. The circular economy can be achieved by utilizing cellulose waste materials to produce hydrogel films as a substitute for plastic wrap. Natural polymer‐based hydrogel films have become a viable option due to their potential for functionalization, biodegradability, and biocompatibility.

Oil palm empty fruit bunches (OPEFB) are lignocellulosic byproducts generated by the palm oil industry and are frequently underutilized. Research has shown that cellulose extracted from OPEFB can be converted into alpha‐cellulose [1]. This alpha‐cellulose can be processed into various derivatives, including microcrystalline cellulose (MCC) [2, 3], carboxymethyl cellulose (CMC) [4–7], and hydrogel films.

The hydrogel film made from a CMC and MCC composite is a viable alternative to plastic wrapping for food. Materials that come into contact with food must be safe; thus, they must employ safe composites and crosslinkers. Hydrogel films based on biomaterials with CMC and MCC formulations are still very limited, as are efforts to use food‐grade crosslinkers such as citric acid. Generally, synthetic PVA polymers are still widely used to enhance the strength of hydrogel films [8–13].

During gelation, creating a stable cellulose hydrogel structure requires a network of three‐dimensional covalent bonds, which is facilitated by the crosslinking process. Crosslinking is essential for producing a hydrogel with excellent absorption characteristics and mechanical strength. Hydrophobizing cellulose‐hydrogel also improves moisture resistance and compatibility with hydrophobic polymer matrices [14, 15]. Modifying cellulose derivatives to be more hydrophobic also reduces their ability to block oxygen. Therefore, a balance of processes and conditions is required to suit each application.

Hydrogels are synthesized through chemical, radiation, or physical crosslinking processes [16]. Crosslinking is essential in hydrogel synthesis to develop hydrogels with a high liquid sorption capacity. The chemicals used for linking agents, although most are toxic, are crucial for the process. Chemical crosslinkers, such as carboxylic acids and carboxylic anhydrides, result in the formation of ‐COOR bonds, esterified components, namely organochlorine compounds, epoxides, and vinyl, resulting in the formation of R–O–R bonds [17]. Other methods include using crosslinkers like divinyl sulfone, poly(ethylene glycol), diglycidyl ether [18], and epichlorohydrin (ECH) [19].

The crosslinking process employed to enhance the strength of hydrogels significantly affects their overall durability. Chemical crosslinking is generally more effective than physical crosslinking, as it utilizes a chemical crosslinker. Since hydrogel products may come into contact with food, it is crucial to identify a food‐grade chemical replacement for smart packaging intended for food applications. There are several studies of CMC‐based hydrogel synthesis using EDGE crosslinkers [20], ECH [21], FeCl_3_ [22], ZnCl_2_ [23], CaCl_2_ [24], sodium trimethaphosphate [25], and citric acid [26–29]. Glutaraldehyde and ECH are commonly used as crosslinkers in hydrogel films. These chemicals are toxic, irritating, and carcinogenic [30–32].

Previous research conducted experiments to examine the synthesis of hydrogels using carboxylic derivatives of succinic acid [29], while [33] indicated that citric acid is a safe and effective crosslinking agent for biomaterials. When formulating hydrogels for food products and for direct contact with food, it is crucial to prioritize the use of safe chemical crosslinkers, including those derived from various carboxylic acid materials, in line with the principles of green chemistry.

Citric acid is a green chemical currently used as a crosslinking agent [34]. The production of hydrogels with strong and high absorption capacity depends on the proper crosslinking process and the suitability of crosslinker agents. Therefore, a special investigation into the crosslinker concentration and crosslinking technique will also be conducted in the hydrogel process using CMC and MCC formulations based on OPEFB cellulose. Some hydrogel products that have been synthesized using citric acid include collagen‐based materials [35], cellulose [36], chitosan [37, 38], polyvinyl alcohol [39], starch [40], and gelatin [41].

Biomaterial‐based hydrogel products are increasingly considered alternatives to synthetic hydrogels, primarily due to concerns about potential toxicity. The development of hydrogels for food packaging offers an exciting opportunity that has yet to be fully explored, particularly for active packaging applications due to their high absorbency properties. Balancing hydrophilic and hydrophobic characteristics ensures the material remains mechanically strong. The effectiveness of the crosslinking method employed is crucial in achieving this balance. Therefore, chemical and physical crosslinking methods should be investigated to produce a hydrogel suitable for smart packaging. Such a hydrogel should be strong enough to resist dissolution upon contact with water while also being sensitive to changes in humidity and pH within the packaging environment.

In addition to chemical crosslinking, the reduction in chemical usage has been achieved through physical techniques such as freeze–thaw and photo crosslinking using UV light and gamma radiation [42]. It is imperative to thoroughly explore the combination of chemical and physical crosslinks to determine the optimal crosslinking process conditions for creating a robust hydrogel film composed of CMC and MCC from OPEFB.

Incorporating citric acid as a green crosslinker necessitates an effective physical crosslinking method to achieve a desirable balance between absorption power and mechanical strength. Intensified crosslinking reduces hydrophilicity and creates a denser hydrogel matrix, decreasing water absorption capacity. The combination of freeze–thaw and heat processes is worth considering as a crosslinking cycle to produce a resilient hydrogel film with high water absorption capacity.

The research selected the formulation of CMC and MCC from OPEFB cellulose using citric acid as a linking agent, which results in a good hydrogel film. CMC is soluble in water and helps with water absorption in hydrogels. MCC is necessary as a filler component to reduce water solubility and increase mechanical strength in hydrogels. Citric acid will be used as a crosslinker due to its carboxylic functional group, which can form bonds with cellulose chains. Additionally, citric acid is an effective crosslinking agent that can address the issues of toxicity and cost in hydrogel production.

## 2. Materials and Methods

### 2.1. Materials

OPEFB (PT Batu Gunung Mulia Putra Agro, Kalimantan Selatan, Indonesia), NaOH (Merck), NaClO_2_ (Clover Chemicals Ltd), glacial acetic acid (Merck), analytical‐grade HCl, isopropanol, monochloroacetic acid (MCA), ethanol, and methanol (Merck), commercial CMC (Sigma‐Aldrich), and distilled water were used as received. Sample preparation employed standard laboratory glassware, a centrifuge, a hotplate stirrer, filtration media, a desiccator, a grinder (Getra IC‐06B, China), and a drying oven (Memmert, Germany). Structural, chemical, morphological, and thermal properties were characterized using XRD (Rigaku MiniFlex with Hypix‐400MF 2D HPAD detector, Japan), ATR‐FTIR (Bruker Alpha, Australia), SEM (FEI Inspect S‐50, Japan), and simultaneous TGA‐DT‐DSC (NEXTA STA, Hitachi STA200 RV, United Kingdom).

### 2.2. Pretreatment of OPEFB Fibers

OPEFB fibers were separated and rinsed with water, followed by surfactant‐assisted cleaning (2%, w/v; 5 h) to remove residual lipophilic components and surface‐bound impurities. After hot‐water rinsing, the fibers were oven‐dried at 60°C for 48 h, reduced to approximately 5 cm in length, and sieved through a 30‐mesh sieve prior to subsequent processing.

### 2.3. Extraction of Cellulose

OPEFB fibers were subjected to two oxidative chlorite bleaching cycles (NaClO_2_, 3.22%, w/v; fiber‐to‐liquor ratio 1:25) under mildly acidic conditions (pH 4.0–4.5, acetic acid) at 75°C ± 5°C for 1 h to disrupt chromophoric lignin structures. The bleached fibers were subsequently treated with 10% (w/v) NaOH at a 1:20 ratio and ambient temperature (30°C) to promote delignification, followed by thorough washing, brief reflux (30 min), and drying at 60°C ± 5°C for 24 h [1].

### 2.4. Synthesis of CMC

Cellulose was alkalized by controlled addition of 30% (w/v) NaOH (cellulose/NaOH = 1:3.36), swollen in isopropanol (1:30.62) at 27°C for 1 h, and subsequently etherified with MCA (cellulose/MCA = 1:1.19) at 45°C for 3 h. The solid product was isolated, solvent‐exchanged with methanol (30‐fold relative to pulp) for 24 h, neutralized with acetic acid, washed with 70% and 96% ethanol to remove byproducts, and dried at 60°C for 24 h prior to grinding [43].

### 2.5. Preparation of MCC

OPEFB‐derived cellulose was subjected to controlled acid hydrolysis using 2.5 N hydrochloric acid at 100°C ± 2°C for 45 min with a solid‐to‐liquid ratio of 1:30 to selectively cleave amorphous domains. The hydrolyzed suspension was subsequently separated, thoroughly washed with distilled water to neutral pH, oven‐dried at 60°C for 24 h, and mechanically pulverized to obtain MCC powder.

### 2.6. Hydrogel Film Preparation

A NaOH/urea/water solvent system (6:4:90, w/w/w; 100 mL) was employed to disperse MCC (3%, w/v), followed by stirring, freeze–thaw treatment (18 h), and centrifugation to obtain a homogeneous cellulose‐rich phase, while a separate 3% (w/v) CMC solution was prepared in water. The MCC and CMC phases were combined at predefined ratios with glycerol (≤ 10%, w/w) and citric acid to induce network formation, homogenized for 2 h, subjected to a second freeze–thaw cycle, and thermally cast at 80°C for 24 h to yield hydrogel films.

### 2.7. Experimental Design

Hydrogel films were constructed by blending CMC and MCC solutions across a compositional window of 100:0–50:50 (w/w) and crosslinked with citric acid (5%–10%) through dissolution‐assisted structuring, freeze–thaw‐induced network development, and thermal curing at 80°C for 24 h.

### 2.8. Characterization of Hydrogel Film

Hydration‐related functionality of the hydrogel films was assessed through water uptake, swelling, gel fraction, and rehydration behavior, while network chemistry, structural organization, morphology, and thermal stability were examined using ATR‐FTIR, XRD, SEM, and TGA–DT–DSC, respectively.

#### 2.8.1. Water Uptake, Swelling, and Gel Fraction

The weighted hydrogel mass is soaked in water at a pH of 7.0 ± 0.5 and in a pH 4.0 acetate buffer for 24 h. After swelling, the gel is wiped and weighed. The sample was oven‐dried at 105°C for 5 h and weighed immediately upon removal. The water uptake is calculated using equation (1), swelling using equation (2), and gel fraction using Equation (3):

(1) water uptake %=Ws−WtWt×100%,

(2) swelling %=WsWt×100%,

(3) gel fraction %=WdWt×100%,

where *W*_*s*_ denotes the mass of the hydrogel in its swollen state, *W*_*d*_ corresponds to the mass of the hydrogel after drying following the swelling experiment, and *W*_*t*_ represents the initial dry mass of the hydrogel prior to testing.

#### 2.8.2. Rehydration Capacity

The dried gel samples were immersed in an aqueous medium at pH 7.0 ± 0.5 for 24 h to allow rehydration. Afterward, excess surface moisture was gently removed, and the swollen mass was recorded. Rehydration capacity was subsequently determined using the corresponding Equation (4):

(4) rehydration %=WrWd×100%,

where *W*_*d*_ represents the postswelling dry mass of the hydrogel, while *W*_*r*_ corresponds to the mass measured after subsequent rehydration.

#### 2.8.3. Fourier Transform Infrared Analysis

Infrared spectra were collected using an ATR‐FTIR instrument (Bruker Alpha, Model 200546) with KBr‐based sample preparation. Data acquisition was carried out at a spectral resolution of 2 cm^−1^ within the 4000–400 cm^−1^ range, and characteristic absorption bands were quantified by peak integration using OriginPro software.

#### 2.8.4. X‐Ray Diffraction

Hydrogel crystallinity was assessed by XRD using Ni‐filtered Cu Kα radiation (30 kV, 30 mA), with diffraction patterns recorded over 2*θ* = 3°–90° (step size 0.02°, scan rate 10° min^−1^) and the crystallinity index (CrI) calculated using the Segal method [44] by Equation (5):

(5) CrI %=I002−IamI002×100,

where *I*_002_ is the intensity of the (002) reflection at 2*θ* ≈ 22.0°–23.0° and *I*_am_ is the amorphous intensity at 2*θ* ≈ 15.0°–17.0°, with d‐spacings and crystallite sizes calculated using Bragg’s law and the Scherrer equation, as follows (6):

(6) L=Kλβ.cosθ,

where *L* denotes the crystallite dimension (nm), *K* is the Scherrer constant (0.94), *λ* represents the X‐ray wavelength (0.15418 nm), *β* corresponds to the full width at half‐maximum (FWHM) of the diffraction peak expressed in radians, and *θ* is the associated Bragg reflection angle.

#### 2.8.5. Thermal Analysis

Thermal behavior of the dried hydrogel samples was characterized by simultaneous TGA–DT–DSC measurements, using sample masses of 3–10 mg. Analyses were conducted over a temperature range of 30°C–550°C at a heating rate of 5°C/min^−1^ under a nitrogen atmosphere flowing at 100 mL/min^−1^.

### 2.9. Data Analysis

Quantitative results were evaluated by analysis of variance (ANOVA) with a confidence level of 95% (*α* = 0.05), and mean comparisons were performed using Duncan’s multiple range test when significant effects were detected. Statistical processing was carried out using IBM SPSS Statistics (v24), while detailed datasets related to functional group analysis, crystallinity, crystallite dimensions, morphological features, and thermal behavior were processed and visualized using OriginPro.

## 3. Results and Discussion

### 3.1. Characterization of CMC

CMC based on cellulose from OPEFB is produced via an alkali treatment and etherification process, with the optimum alkali treatment stage carried out at a cellulose‐to‐NaOH 30% ratio of 1:3.36 and a cellulose‐to‐isopropanol maceration medium ratio of 1:30.6. The etherification stage was carried out at a cellulose‐to‐MCA ratio of 1:1.19. The produced CMC exhibited a 1.28 degree of substitution (DS), 31.93% carboxymethyl, and 0.25% NaCl impurities. A DS greater than 1.0 implies good water solubility and could boost compatibility with hydrogel films. The DS of CMC was confirmed using SEM‐EDX. A DS of 1.20 was determined using the percentage of Na calculated from SEM‐EDX analysis.

The comparison was conducted using functional group patterns obtained by FTIR and crystallinity analysis by XRD on CMC OPEFB and commercial CMC. OPEFB CMC and commercial CMC showed similar patterns. Both commercial CMC and CMC OPEFB showed sharpness at 1579 cm^−1^, reflecting an enhancement in carbonyl (C=O) functional groups as a result of carboxymethyl substitution in cellulose. The degree of crystallinity of CMC OPEFB was 40.18%, which was slightly lower than that of commercial CMC (49.72%). Overall, CMC OPEFB was comparable to commercial CMC.

### 3.2. Characterization of MCC

The MCC obtained from OPEFB cellulose used in this hydrogel formulation was processed through 2.5 N HCl acid hydrolysis for 45 min. MCC demonstrated pronounced hydration behavior, with swelling and water absorption capacities of 5.03 ± 0.26 and 4.03 ± 0.26 g g^−1^, respectively. The CrI of MCC from OPEFB is 88.89% ± 4.76%, which is comparable to the CrI of commercial MCC (89.52%). Similarly, the crystallite size of OPEFB MCC at 4.23 nm is slightly larger than that of commercial MCC at 3.94 nm. MCC served as a structural filler that balances hydration performance with enhanced crystallinity, supporting the mechanical stability of the resulting hydrogel film.

### 3.3. Characterization of Hydrogel Film

This hydrogel film was formulated using CMC and MCC sourced from OPEFB’s cellulose. While beneficial, the natural hydrogen bonding between CMC and MCC is insufficient to yield a robust hydrogel film. A crosslinking agent enhances the film’s strength and structural integrity. In this case, citric acid is employed as a green chemistry alternative, serving as an effective bridging agent that links the CMC and MCC molecules together. Building on the role of MCC in balancing hydration behavior and structural reinforcement, a targeted physical treatment was subsequently introduced to further optimize the organization and performance of the hydrogel network. This involves a carefully controlled stage of the freezing and thawing phase, which helps reorganize the molecular structure and increase the resilience of the hydrogel film. Following this treatment, the mixture is cast at elevated temperatures, resulting in a strong, cohesive hydrogel film that harnesses the benefits of its natural components and the crosslinking process.

A polymer solution undergoes significant alteration during the freeze–thaw phase due to physical crosslinking caused by the crystallization process. Initially, the polymer chains contain hydroxyl groups that interact with surrounding water molecules to form hydrogen bonds. These bonds are essential for maintaining the solution’s structure.

As the polymer solution is frozen, the temperature drop initiates the formation of ice crystals. This crystal growth acts as a network of crosslinks that physically connect the polymer chains, creating a more structured and rigid arrangement. The crystals effectively bind the polymer chains, increasing viscosity and altering the material’s properties. Upon thawing, the temperature rises, and the ice crystals melt, releasing the constraints on the polymer chains [45]. This phase allows the polymer chains to relax and regain their original, flexible state. The ability of the polymer to transition between these states, from a rigid, crosslinked structure during freezing to a more fluid state upon thawing, is a key characteristic that can be utilized in various applications, including drug delivery systems, tissue engineering, and other innovative materials technologies.

**FIGURE 1:**
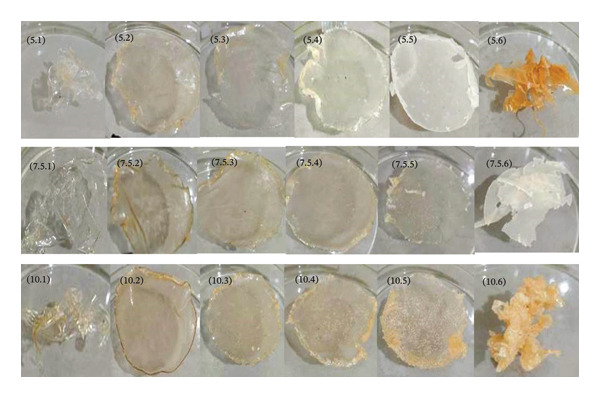
Hydrogel film at different concentrations of citric acid (5%, 7.5%, 10%) and various proportions of CMC:MCC (100:0, 90:10, 80:20, 70:30, 60:40, 50:50).

Figure 1 illustrates that a 100% CMC concentration yields an elastic hydrogel film that can be easily torn. As the MCC concentration increases to 50%, the hydrogels become clay‐like, brittle, or fragile. Increasing the citric acid concentration in hydrogel films makes them more durable and rigid, but less elastic, which makes them harder to remove from molds. Citric acid serves as a crosslinking agent, bonding molecules in the CMC:MCC formulation. More crosslinking creates a denser network, reducing the mobility of polymer chains and resulting in stiffer materials [46].

Interchain crosslinking generates a three‐dimensional polymer network that immobilizes the surrounding solvent, resulting in gel formation [47]. An increased density of hydrophilic moieties (–OH, –COOH, and –NH_2_) enhances both intra‐ and intermolecular hydrogen bonding among polymer chains, thereby promoting the development of a three‐dimensional network that can retain water.

Hydrogels composed of CMC, MCC, glycerol, and citric acid as a crosslinker exhibit a yellowish‐brown appearance. This is assumed to be the result of numerous chemical reactions catalyzed by heat and acid [48]. Citric acid crosslinks cellulose derivatives via esterification [49, 50]. Heating reduces water and forms ester bonds.

(7) cellulose–OH+citric acid–COOH⟶cellulose–O–CO–citric+H2O.

Citric acid dehydrates in high temperatures and acidic conditions, forming aconitic acid, itaconic acid, or other organic components. These compounds will polymerize or undergo further oxidation, producing darker‐colored byproducts such as mild caramelization [51, 52].

Similarly, glycerol heated in acidic conditions can be dehydrated to produce acrolein. Acrolein and its oxidation products range in color from yellow to brown, which can react with citric acid or hydroxyl groups in cellulose to generate a more intense color. This hydrogel casting process uses a temperature of 80°C for 24 h, which also allows the breakdown of glycosidic linkages, resulting in the synthesis of furfural or hydroxyfurfural, which has a yellow to brown color and strongly absorbs visible light [53].

(8) glycerol⟶acidheatacrolein CH2=CH–CHO+2H2O.

#### 3.3.1. Water Uptake, Gel Fraction, and Swelling

The water uptake measurement determines the amount of water absorbed under specific conditions. Water uptake measurements were taken at neutral and acidic pH (pH 4). At alkaline pH, the hydrogel dissolved. The amount of water absorbed is directly related to the level of hydrogel swelling.

The higher water absorption leads to increased swelling. A negative water absorption rate indicates that the hydrogel matrix dissolved in the immersion medium. Treatment 5.3 exhibited the highest water absorption level. However, in this case, the hydrogel may have dissolved due to the suspected crosslink with a high level of hydrophilic capacity at 20% MCC in the formula, which is at the maximum limit for maintaining hydrogel rigidity, causing it to be in a state of partial dissolution.

Table 1 indicates that the gel fraction of the hydrogel film decreases as the ratio of MCC to CMC in the hydrogel formulation increases. A higher ratio of MCC weakens the hydrogen bonds in the hydrogel, causing more gel to dissolve upon contact with water. Regardless of the concentration of citric acid, the highest gel fraction occurs when the ratio of CMC to MCC is 100:0. This suggests that CMC serves as the backbone in the hydrogel formula, and with the appropriate crosslinking process, the crosslinking in the composition of CMC becomes increasingly intense due to its hydrophilic nature. The presence of MCC, with its dominant crystalline structure, reduces the crosslinking in the hydrogel. Some hydrogel films showed negative values, indicating a loss of hydrogel mass due to partial dissolution. The swelling response of CMC‐based hydrogel films is primarily driven by carboxymethyl substituents (–CH_2_–COOH) that enhance polymer–water affinity, thereby supporting high levels of water absorption and retention. Swelling behavior is further modulated by structural and formulation variables such as substitution degree, network crosslinking, pH conditions, and filler content [54].

**TABLE 1:** Gel fraction and water uptake of hydrogel film at pH 7 and pH 4.

Cons. of citric acid (%)	CMC:MCC	Gel fraction (%)		Water uptake (%)	
		pH 7	pH 4	pH 7	pH 4
5	100:0	11.34 ± 0.32^hi^	12.20 ± 0.01^j^	64.70 ± 3.27^b^	51.74 ± 3.44d^e^
	90:10	9.87 ± 0.26^fg^	11.59 ± 1.12^hi^	222.72 ± 9.32^c^	340.35 ± 18.56^g^
	80:20	5.89 ± 0.03^c^	9.20 ± 0.90^def^	603.02 ± 26.98^e^	94.42 ± 2.98^f^
	70:30	3.45 ± 0.29^b^	5.83 ± 0.23^b^	130.38 ± 4.78^b^	−2.84 ± 1.35^b^
	60:40	0.00^a^	3.82 ± 0.10^a^	−82.35 ± 8.16^a^	−28.71 ± 4.45^b^
	50:50	0.00^a^	3.27 ± 0.63^a^	−80.18 ± 4.86^a^	−56.80 ± 5.18^a^

7.5	100:0	11.32 ± 0.17^hi^	11.92 ± 0.41^i^	61.38 ± 2.07^b^	40.43 ± 4.04cd^e^
	90:10	10.54 ± 0.65^b^	11.04 ± 0.08^ghi^	96.00 ± 6.47^b^	53.85 ± 8.23d^e^
	80:20	10.58 ± 0.35^gh^	10.28 ± 0.28^fgh^	125.69 ± 6.79^b^	57.34 ± 6.60^e^
	70:30	7.34 ± 1.03^d^	9.36 ± 0.20^def^	216.73 ± 5.39^c^	49.71 ± 2.12^de^
	60:40	5.07 ± 0.22^c^	8.55 ± 0.52^cde^	338.62 ± 4.12^d^	29.43 ± 3.54^cd^
	50:50	4.74 ± 0.21^c^	7.51 ± 0.35^c^	103.15 ± 5.28	4.72 ± 0.75^b^

10	100:0	12.31 ± 1.08^i^	14.53 ± 1.18^k^	75.81 ± 5.38^b^	45.71 ± 7.25^de^
	90:10	8.27 ± 0.90^de^	9.83 ± 0.56^efg^	80.36 ± 5.33^b^	46.62 ± 5.91^de^
	80:20	7.63 ± 0.71^d^	9.99 ± 0.33^efg^	102.22 ± 2.24^b^	56.69 ± 4.97^e^
	70:30	7.57 ± 0.21^d^	7.89 ± 0.19^cd^	335.07 ± 8.53^d^	52.76 ± 0.78^de^
	60:40	8.97 ± 0.24^ef^	7.90 ± 0.36^cd^	94.88 ± 7.24^b^	17.81 ± 1.98^bc^
	50:50	5.71 ± 0.04^c^	7.08 ± 0.39^bc^	87.56 ± 3.05^b^	−5.28 ± 1.09^b^

*Note:* Different letters in the same column indicate significantly different treatments (*p* ≤ 0.05). (−) indicates dissolution and loss of mass.

The gel fraction is a crucial indicator of the number of crosslinks in the hydrogel. A higher number of crosslinks results in a more resistant hydrogel, increasing the maintainable gel fraction. Our findings show that at pH 7, the gel fraction is at its lowest with a 5% citric acid concentration (5.09%). Additionally, we observed that the difference in gel fraction between 7.5% citric acid (8.27%) and 10% (8.41%) was insignificant. Notably, the gel fraction was relatively higher in acidic conditions than at pH 7. While increased citric acid concentration as a crosslinking agent tends to decrease the gel fraction, the difference between 7.5% citric acid (9.77%) and 10% citric acid (9.54% was relatively indistinguishable.

Figure 2 illustrates the swelling behavior in the hydrogels for each treatment. At a 5% citric acid concentration, the gel remains firm for up to 24 h in treatments 5.1 and 5.2 (CMC:MCC 100:0 and 90:10). However, in treatment 5.3 (80:20), the gel expands and dissolves within the first 2 h. The maximum swelling occurs within the first 2 h of immersion, after which the swelling tends to stabilize, or the gel may even dissolve. Gel dissolution typically occurs in treatment 7.5.4, which involves a 7.5% citric acid concentration (CMC:MCC 70:30), and in treatment 10.5 (10% citric acid, CMC:MCC 60:40). As a bio‐derived and nontoxic crosslinker, citric acid induces esterification reactions between its carboxyl groups and the hydroxyl moieties of CMC under thermal curing conditions, generating a densified polymer network that strengthens water stability and mechanical integrity [55].

FIGURE 2Swelling pattern per 2 h in each hydrogel film: (a) 5.1–5.2, (b) 7.5.1–7.5.4, and (c) 10.1–10.5.

**Figure:**
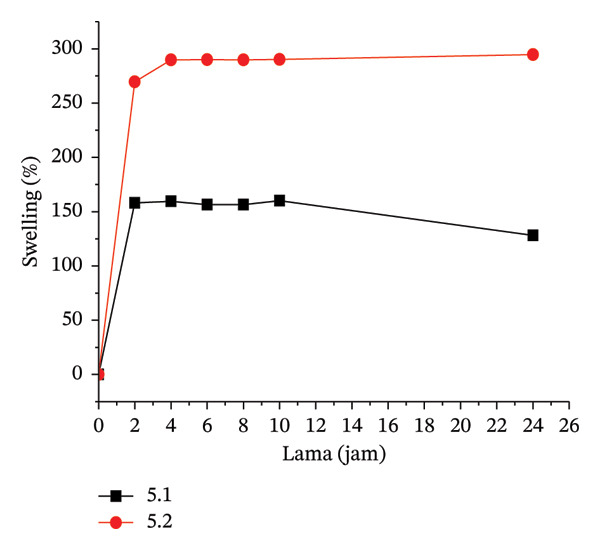
(a)

**Figure:**
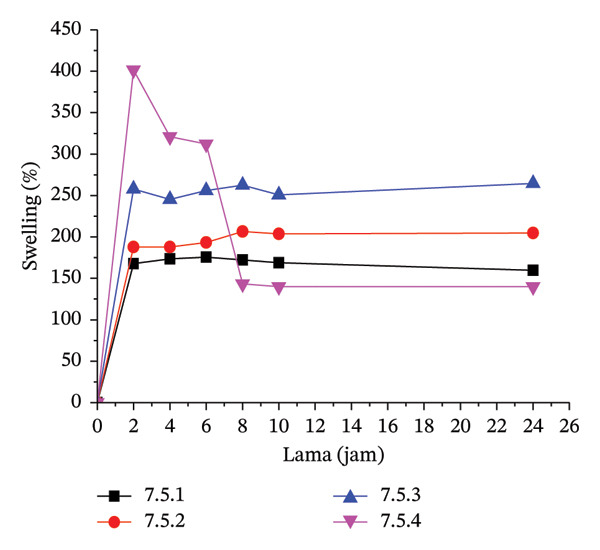
(b)

**Figure:**
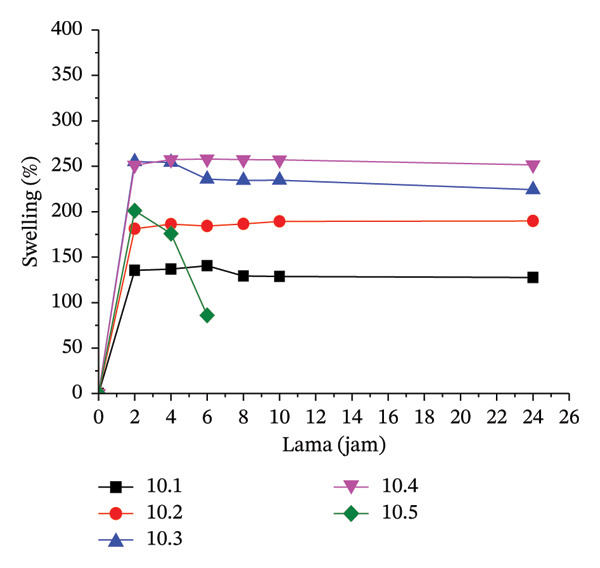
(c)

The hydrogel network is established through a combination of reversible hydrogen bonding among cellulose hydroxyl groups and permanent covalent junctions introduced by citric‐acid‐mediated ester linkages. Upon hydration, hydrogen bonds are partially disrupted, which can weaken the network in the absence of covalent stabilization; however, ester crosslinks remain intact in aqueous environments, thereby preserving the structural integrity of the hydrogel. These covalent junctions restrict excessive chain mobility and swelling, thereby maintaining network cohesion under wet conditions. The extent of mechanical robustness is directly associated with crosslink density, which develops initially during freezing and is subsequently reinforced during thermal curing at 80°C for 24 h, yielding progressively stronger hydrogel matrices as crosslink concentration increases.

Hydrogels typically have a firmer texture at 10% citric acid concentrations than at 7.5% or 5%, likely due to more crosslinking. The swelling level at 10% citric acid concentration is not significantly different from that at 7.5%. The number of carboxylic groups does not completely correlate with the occurrence of crosslinking. The crosslinking mechanism involves heating citric acid, which dehydrates to form a cyclic anhydride that reacts with the polymer. The carboxylic acid will react with the hydroxyl group of cellulose through esterification; thus, further esterification with hydroxyl on other cellulose will produce crosslinking between cellulose chains [56].

Barbucci et al. [57] provided elucidation of the bifunctional role of carboxyl moieties in regulating hydrogel hydration behavior. In their protonated form (–COOH), these groups promote intermolecular hydrogen bonding, which restricts polymer chain expansion and limits water uptake. Conversely, deprotonation to the carboxylate state (–COO^−^) introduces electrostatic repulsion between chains, facilitating network expansion and enhanced water absorption.

Table 2 shows that the rehydrated dry gel fraction can still swell again. The gel can still absorb a certain amount of water, with a percentage more significant than the swelling ability of the initial hydrogel film. The hydrogel treatment 5.3 can absorb water of 1605.06% of the dry gel fraction or 16 times the initial weight, and the hydrogel treatment 7.5.6 can rehydrate 1359.44% of the dry gel fraction. A 10% citric acid concentration is a relatively smaller rehydration level than 5% and 10% citric acid concentrations. It is suspected that the crosslinks formed at a 10% citric acid concentration are more pronounced, resulting in a relatively low water absorption ability in the dry gel fraction compared to the others.

**TABLE 2:** Rehydration capacity of dry gel (%) of hydrogel films.

CMC:MCC	Conc. of citric acid (%)		
	5	7.5	10
100:0	299.09 ± 60.59	190.09 ± 11.24	225.00 ± 15.71
90:10	466.96 ± 30.41	269.26 ± 38.23	242.25 ± 51.09
80:20	1605.06 ± 41.21	424.72 ± 69.24	325.63 ± 47.71
70:30	0.00	650.00 ± 70.71	531.67 ± 29.63
60:40	0.00	762.16 ± 65.81	389.84 ± 29.90
50:50	0.00	1359.44 ± 32.92	683.72 ± 19.73

#### 3.3.2. Tensile Strength

Tensile testing revealed that the mechanical resistance of the hydrogel films is strongly influenced by both polymer composition and crosslink density. Films composed entirely of CMC (100:0) with 5% citric acid exhibited the lowest tensile strength, reflecting limited crystallinity and network reinforcement. Incorporation of MCC at an 80:20 ratio slightly reduced tensile strength compared with the 90:10 formulation but increased strain at maximum force, consistent with improved chain packing and higher crystallinity. Higher citric acid content (10%) enhanced crosslink density, which restricted polymer chain mobility and resulted in lower strain at maximum force, demonstrating that mechanical elasticity is directly modulated by the combined effects of crystallinity and covalent crosslinking.

The highest tensile strength was achieved with a CMC:MCC ratio of 50:50, at 0.600 ± 0.002 MPa, using a 5% citric acid concentration. In comparison, the 90:10 ratio yielded a strength of 0.303 ± 0.010 MPa. At a 7.5% citric acid concentration, the 70:30 ratio achieved a tensile strength of 0.542 ± 0.060 MPa, while the 80:20 ratio reached 0.545 ± 0.002 MPa at a 10% concentration. A distinct interaction pattern emerges at each crosslinker concentration with the presence of MCC as a filler (Figure 3). In CMC:MCC 80:20, the tensile strength is considerably high at citric acid concentrations of 7.5% and 10%, while in CMC:MCC 50:50, citric acid concentrations of 5% and 7.5% provide high tensile strength. The tensile strength is a synergistic effect of MCC and citric acid in filling the hydrogel matrix and crosslinking within it.

FIGURE 3Tensile strength and strain at *F* max of the hydrogel film.

**Figure:**
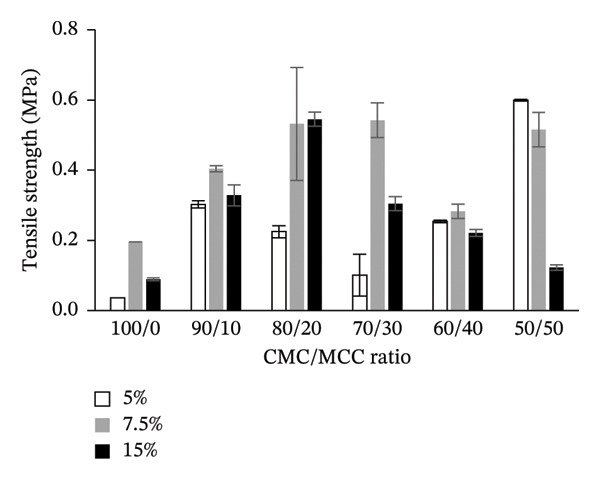
(a)

**Figure:**
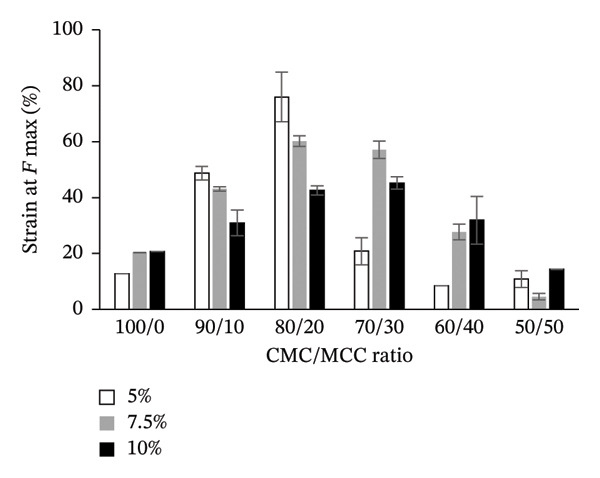
(b)

Each concentration of citric acid utilized as a crosslinking agent produced a distinct pattern in the maximum force‐strain values. The 80:20 ratio of CMC to MCC demonstrated the highest maximum strain values, reported at 75.99% ± 1.87% for a 5% citric acid concentration and 60.22% ± 1.88% for a 7.5% concentration. Conversely, at a 10% citric acid concentration, the maximum strain value for the 80:20 ratio decreased to 42.59% ± 1.72%. The strain at F max tended to decrease as the MCC‐to‐CMC ratio increased, particularly when the filler content exceeded 20%. This occurred as a result of MCC’s crystallinity decreasing the elasticity of the hydrogel film.

Low crosslinks will cause the hydrogel film to lose its integrity; however, at a 10% citric acid concentration, the hydrogel film becomes more rigid. This correlates with the density of crosslinks that form, thereby reducing the mobility of polymer chains and decreasing the free volume within the hydrogel structure. Freeze–thaw treatment and casting at 80°C for 24 h will produce strong crosslinks, making the hydrogel harder and increasing its resistance to tensile and stretching forces.

Hydrogel films with a citric acid concentration of 10% and MCC filler above 30% tend to produce hard and stiff hydrogel films. This is thought to be due to the strong hydrogen bonding interactions between CMC and citric acid, which reduces the expansion and relaxation capacity of the polymer [58]. The more the MCC, the greater the increase in structural density and crystallinity, which reduces the elasticity of the hydrogel film. The functional characteristics of hydrogel films made from CMC are significantly influenced by the incorporation of MC. Due to its rigid crystalline structure, MCC enhances the polymer matrix’s strength and promotes strong intermolecular hydrogen bonding, leading to increased mechanical strength in the films. However, this enhancement may also result in reduced flexibility [59]. Increasing the crosslink agent concentration will increase the crystallinity of the hydrogel and decrease its elasticity, as indicated by the decreased stretch break caused by the suppression of molecular chain mobility and orientation during stretching [60].

#### 3.3.3. Infrared Spectroscopy

The synthesis of the hydrogel film involves using citric acid as a crosslinking agent. It is believed that the carboxyl molecules in citric acid effectively crosslink with CMC and MCC molecules. In Figure 4(a), the broad peak at 3303 cm^−1^ indicates the presence of hydroxyl groups binding to hydrogen, while the peak at 1021 cm^−1^ is related to C–O stretching vibrations in the C–O–C group [61, 62]. Additionally, the peak at 1578 cm^−1^ indicates the presence of carboxyl in CMC, and the peak at 1705 cm^−1^ was caused by C=O stretch vibrations in the hydroxyl group, confirming the chemical crosslinking of CMC and citric acid.

FIGURE 4FTIR profile of hydrogel film: (a) differences of wavenumbers per treatment and (b) differences in peak wave numbers of treatments 5.2 and 5.3.

**Figure:**
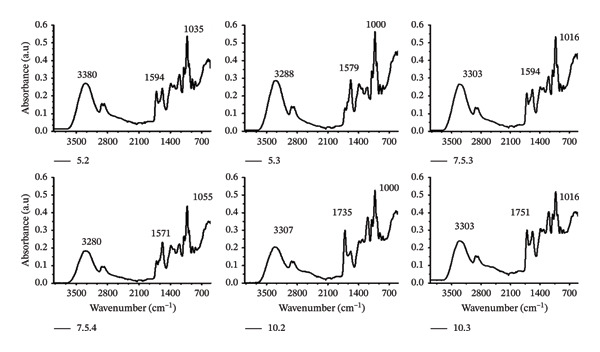
(a)

**Figure:**
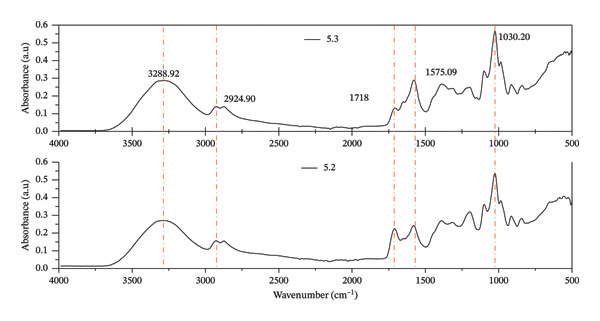
(b)

Analytical evidence of crosslinking in FTIR is indicated by the intensity of the ester C=O peak and the reduced intensity of the –OH band [63]. Esterification of cellulose derivatives with citric acid increases crosslink density, a process that depends on the concentration of citric acid. Increased crosslinking reduces network mobility and increases film stiffness [64, 65].

Increasing citric acid concentration enhances esterification, crosslinks CMC films, and improves water resistance and tensile strength up to the optimum citric acid concentration. The performance of citric acid crosslinking at the optimum concentration is evident in the formation of esters, as well as in the swelling phenomenon and mechanical strength [66]. Higher concentrations will reduce mechanical integrity because they exceed the plasticity limit [66]. The multifunctional ability of the carboxyl group in citric acid, in esterification with the hydroxyl groups of polysaccharides, correlates with increasing citric acid content and the intensity of the curing process. Crosslinking will become denser, but swelling capacity will decrease [67].

The treatment of 5.2, which has a higher crosslink density than 5.3, yields significantly improved mechanical strength. Treatment 5.3 absorbs substantial water but degrades, resulting in notable hydrogel formation. In treatment 10.3, the balance between the peak wave numbers at 1705 and 1578 cm^−1^ strongly correlates with resistance to solubility and excellent swelling ability. The notable contrast is evident between treatments 7.5.1 and 7.5.6. The absence of MCC on 7.5.1 leads to a sharpening of the wave number at 1723 cm^−1^, whereas in 7.5.6, with a CMC‐to‐MCC ratio of 50:50, the wave number sharpening occurs at 1583 and 1384 cm^−1^.

The changes in wave number dominance in treatments 5.2 and 5.3 are explained in Figure 4(b). In treatment 5.2, a strong peak is observed at a wavenumber of 1723 cm^−1^, indicating the protonated state of the carboxyl group (COOH) and its lower absorption properties. Treatment 5.3 exhibits dominance at 1575 cm^−1^, indicating the presence of a carbonyl group (COO–). This suggests that ionized carboxylic groups more readily absorb water into the hydrogel matrix. As a result, the mechanical strength of the hydrogel film will be weakened by the amount of ionized carboxyl, leading to the disintegration of the structure.

#### 3.3.4. X‐Ray Diffraction

The hydrogel film containing MCC filler, along with the crosslinking process, affects the crystallinity of the hydrogel. Notably, the maximum intensity for the 2*θ* is observed in the range 20°–22°. Hydrogel films with 5% and 7.5% citric acid exhibit a lower slope in the 2*θ*_max_ intensity when the ratio of CMC and MCC is 100:0. In contrast, at a 10% citric acid concentration, the 2*θ*_max_ intensity demonstrates a slope at both CMC:MCC 100:0 and a CMC:MCC ratio of 50:50. Figure 5 illustrate evidence that treatments 5.2, 7.5.2, and 10.2 tend to have a more intense sharpening effect than those at the same citric acid concentration. The trend in the proportion of CMC:MCC 100:0 shows lower intensity than the others. Table 3 shows that the hydrogel film exhibits a significant CrI. When the proportion of MCC in the formulation is increased by 5% citric acid, the CrI also increases. However, this trend is inconsistent at 7.5% and 10% citric acid concentrations.

FIGURE 5XRD profiles of hydrogel films: (a) 5.1–5.6, (b) 7.5.1–7.5.6, and (c) 10.1–10.6.

**Figure:**
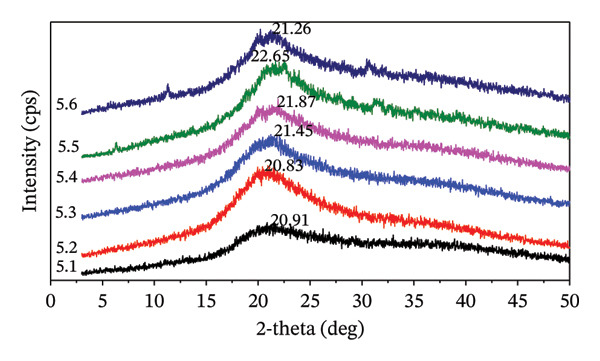
(a)

**Figure:**
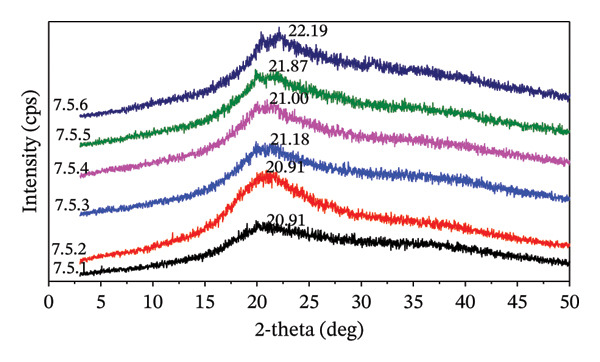
(b)

**Figure:**
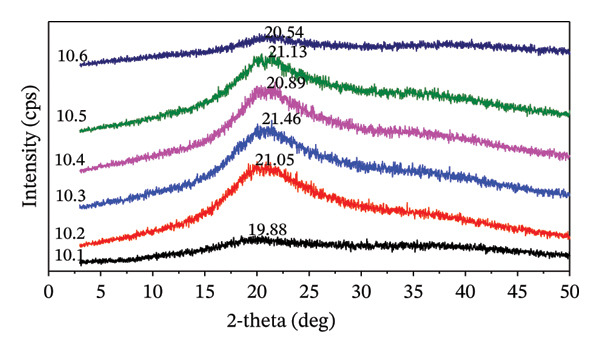
(c)

**TABLE 3:** Crystallinity index (CrI) of hydrogel film.

Treatments	2*θ* (*I*_002_)	2*θ* amorf	CrI
5.1	37.00	20.53	5.49
5.2	20.85	35.96	15.00
5.3	20.63	41.37	30.36
5.4	19.94	36.73	49.05
5.5	11.92	16.90	65.06
5.6	21.40	19.82	76.07
7.5.1	36.38	20.64	27.16
7.5.2	20.99	35.96	54.20
7.5.3	21.41	34.83	30.49
7.5.4	20.76	36.81	91.68
7.5.5	19.77	35.60	98.92
7.5.6	20.36	36.66	54.64
10.1	37.40	19.78	46.75
10.2	20.71	35.68	44.04
10.3	20.08	21.55	28.94
10.4	22.20	18.00	33.09
10.5	20.80	18.89	64.67
10.6	36.00	38.50	75.33

The data shows that 5% citric acid exhibits a pattern of increasing crystallinity consistent with an increase in MCC, suggesting that crosslinking occurs effectively and results in a stronger molecular structure. The resulting hydrogel will be stiff, brittle, and less flexible as CrIs. In hydrogel films with a concentration of 7.5%, the variability in crystallinity is quite large, with 7.5.4 and 7.5.5 having high crystallinity. This treatment resulted in highly ordered structures that were rigid and glassy. Meanwhile, a citric acid concentration of 10% produced a nonlinear effect on the crystallinity of the hydrogel, with optimal crystallization likely occurring at high MCC formulations, namely 10.5 and 10.6.

Hydrogels with CMC, MCC, and citric acid as crosslinkers exhibit an increase in CrI when chain mobility decreases or under specific conditions that enable the reorganization of molecules within them into a more orderly arrangement. Meanwhile, the amorphous area is generally more sensitive to hydration, plasticizers, and network mobility.

A reduced CrI corresponds to a more flexible hydrogel network, whereas higher crystallinity enhances mechanical strength. In hydrogel films containing 7.5% and 10% citric acid, the CrI is elevated due to the increased MCC content and the formation of covalent crosslinks with citric acid.

The mechanical strengthening often correlates with an increase in crystalline/frozen domains. An intensive freeze–thaw process will produce a hydrogel with high crystallinity, thereby increasing its strength and stiffness [68]. The presence of MCC with high crystallinity in the hydrogel formula will correlate with the stiffness of the hydrogel; however, it will also render the hydrogel brittle. Similarly, cellulose nanocrystal‐based films with high crystallinity exhibit strong but brittle properties, while films with low crystallinity are more flexible [69]. The presence of crystalline and regular areas, or strong hydrogen bonds, increases mechanical strength and hardness [70].

Treatment of 5.6 produced a very stiff and brittle hydrogel film, while 7.5.4 and 7.5.5 produced an intact and tenacious hydrogel film. The high crystallinity in these treatments still produces a hydrogel film that dissolves when macerated. This suggests that the CrI of the hydrogel is not directly correlated with the crosslinking or swelling level of the hydrogel films. The measured crystallinity illustrates the presence of MCC crystallinity and the excess of citric acid as a crosslinker agent.

Crystallinity tends to increase with increasing concentrations of citric acid as a crosslinker and with increasing ratios of MCC to CMC. The esterification reaction causes the formation of crosslinks between MCC, CMC, and citric acid.

According to Sannino et al. [17], esterification of OH functional groups of CMC/MCC with cyclic anhydride intermediates will trigger the formation of new carboxylic acid units as a component of new intramolecular formation between anhydride and new carboxylic acid units. The crosslinking process typically occurs under dry conditions and requires higher temperatures for the reaction to proceed. Therefore, the more intensive the crosslink process is at high temperatures, the higher the crystallinity of the hydrogel will be, as more hydrogen bonds will form.

#### 3.3.5. Morphology of Hydrogel Film

**FIGURE 6:**
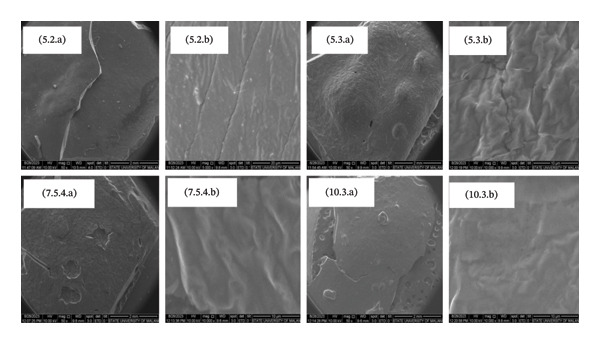
Surface morphology of hydrogel at magnification (a) 50x and (b) 1000x in each treatment 5.2, 5.3, 7.5.4, and 10.3 before swelling.

The surface morphology of the hydrogel film in several treatments exhibited differences (Figure 6). Treatment 5.2, which utilized a 90:10 CMC:MCC concentration with 5% citric acid, exhibited a smoother surface than the other treatments. Treatment 5.3 (CMC:MCC 80:20 with 5% citric acid) exhibited more intense bubbling fractures on the surface and a rougher surface at 100,00x magnification compared to treatments 7.5.4 and 10.3.

In all these treatments, 5.2, 5.3, 7.5.4, and 10.3 could absorb water properly, with the highest absorption capacity in 5.3. In the treatments of 5.2, 7.5.4, and 10.3, the material can absorb water while maintaining its mechanical strength, resulting in a surface that appears sturdier and smoother. In contrast, 5.3 exhibits more pronounced bubbling. Treatment 5.3 features a more porous and rough surface compared to treatment 5.2, which contributes to its greater ability to absorb water and expand more rapidly. The roughness of the surface also enhances its hydrophilicity. In contrast, Treatments 5.2, 7.5.4, and 10.3 exhibit smoother surface characteristics, indicative of a more compact crosslink structure. This results in reduced swelling and increased mechanical strength. These findings align with previous research indicating that rough surface shapes serve as a mechanism for enhancing water absorption capacity [48, 58, 59].

Figure 7 illustrates the hydrogel after swelling. Treatment 5.2 displays a surface fracture due to bubbling caused by water absorption, while treatment 5.3 exhibits a smooth surface. Treatments 5.2, 5.3, 7.5.4, and 10.3 have the most notable water absorption capacities, with treatment 5.3 having the highest capacity. Despite bubbling in treatment 5.3, treatments 5.2, 7.5.4, and 10.3 can absorb water while retaining the mechanical strength of the structure, resulting in a more robust and smooth surface appearance. Hydrogel, after swelling, as shown in 5.2, exhibits surface fracture due to bubbling caused by water absorbed into the matrix. Meanwhile, 5.3 has a smooth surface because the hydrogel 5.3 decays, not dissolves. Therefore, when dried, water in the matrix is easily released, and the hydrogel surface area is effectively reduced.

FIGURE 7Surface morphology of dried hydrogel film in treatments (a) 5.2 and (b) 5.3.

**Figure:**
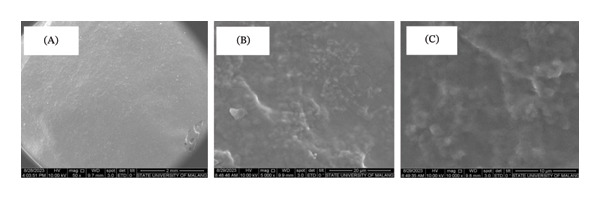
(a)

**Figure:**
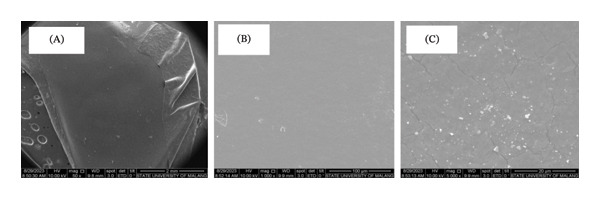
(b)

#### 3.3.6. Thermal Stability of Hydrogel Film

The thermal stability test is designed to assess the resistance of hydrogels to temperature changes by evaluating their mass loss within a specific temperature range. As shown in Figure 8(a), the mass change patterns for treatments 5.2 and 5.3 are relatively similar. The reduction in hydrogel mass within the temperature range of 30°C–250°C is slower compared to the range of 250°C–350°C (Figure 8(b)). Table 4 shows the peak decomposition temperature for hydrogel treatment 5.2, which was recorded at 295.30°C, with a mass loss rate of 354.91 μg/min. In contrast, treatment 5.3 exhibited a maximum decomposition temperature of 287.85°C, accompanied by a mass loss rate of 350.67 μg/min.

FIGURE 8Pattern of (a) TG and DTG profile, and (b) TG and DTG analysis of treatments 5.2 and 5.3.

**Figure:**
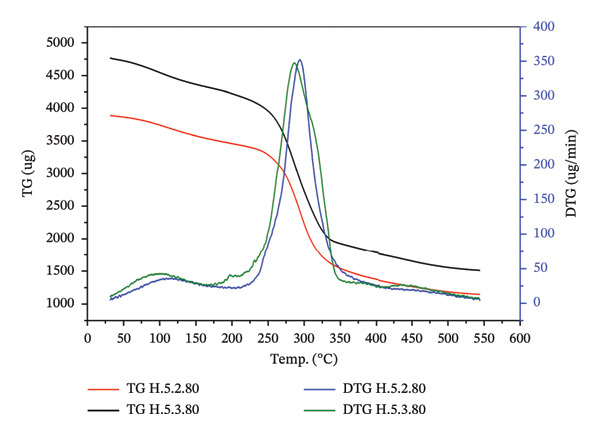
(a)

**Figure:**
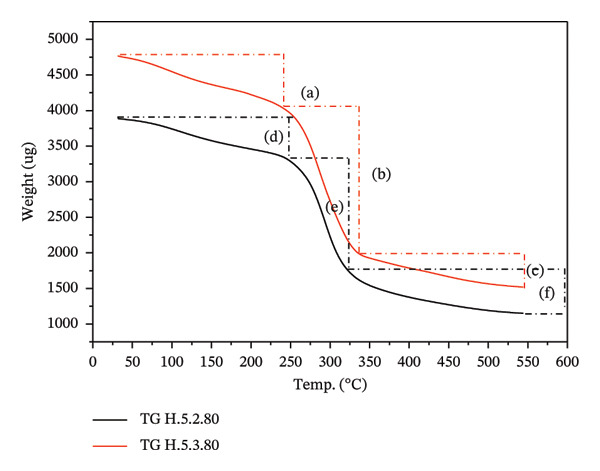
(b)

**TABLE 4:** TG profiles in treatments 5.2 and 5.3.

5.2.80				5.3.80			
**Temp (°C)**		**Weight (μg)**		**Temp (°C)**		**Weight (μg)**	

T1	33.27	W1	3884.39	T1	30.5	W1	4766.61
T2	247.20	W2	3329.47	T2	242.51	W2	4038.29
T3	323.14	W3	1775.29	T3	337.85	W3	1968.20
T4	544.75	W4	1159.68	T4	546.89	W4	1504.39
ΔT_1-2_	213.93 (+643.01%)	ΔW_1-2_ (d)	−554.92 (−14.29%)	ΔT_1-2_	212.01 (+695.11%)	ΔW_1-2_ (a)	−728.32 (−15.28%)
ΔT_2-3_	75.94 (+30.72%)	ΔW_2-3_(e)	−1554.18 (−46.68%)	ΔT_2-3_	95.34 (+39.31%)	ΔW_2-3_ (b)	−2070.09 (−51.26%)
ΔT_3-4_	221.61 (+68.58%)	ΔW_3-4_(f)	−615.61 (−34.68%)	ΔT_3-4_	246.00 (+61.87%)	ΔW_3-4_ (c)	−463.87 (−23.57%)

Mass loss below 100°C was attributed to the evaporation of water. The mass loss occurring between 100°C and the initial decomposition temperature was related to the evaporation of both water and glycerol. Significant temperature increases, up to 250°C, are believed to release volatile compounds and some water from the hydrogel matrix. In the temperature range of 250°C–350°C, the hydrogel undergoes decomposition, resulting in a substantial decrease in mass. The mass loss becomes minimal above 350°C and remains constant up to 550°C. Treatment 5.2 resulted in a residue of 29.58%, while treatment 5.3 yielded a residue of 31.85%.

**FIGURE 9:**
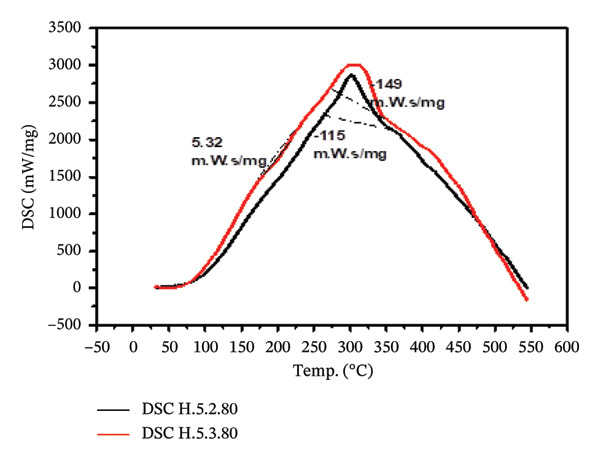
DSC profiles of treatments 5.2 and 5.3.

Figure 9 illustrates the scanning calorimetry profiles for treatments 5.2 and 5.3. The lines of the two profiles are nearly overlapping, indicating that the energy uptake during crystallization in treatment 5.3 is slightly higher than in treatment 5.2. Specifically, the crystallization process in treatment 5.3 released an energy amounting to 149 m.W.s/mg, while treatment 5.2 released 115 m.W.s/mg. This observation is further supported by the scanning morphology results in Figure 6, which show that the dry hydrogel from treatment 5.3 has a smoother surface compared to treatment 5.2. This smoother surface implies a higher level of crystallinity in treatment 5.3, as confirmed by the XRD results.

### 3.4. Prospects of Hydrogel Film for Replacing Plastic Wrapping

Hydrogel films, as a thin layer of hydrogel material, easily absorb environmental moisture. If applied to fresh fruit and vegetable products that respire, it is hoped that a certain amount of exudate moisture from respiration can be absorbed, allowing the environmental conditions of the packaging to be controlled properly. In its function as a wrapping, the hydrogel film must be conditioned to achieve a hydrophilic equilibrium, allowing it to absorb moisture while maintaining hydrophobicity, thereby ensuring the hydrogel film remains in a mechanically solid structure and does not dissolve. It can stick well as a good wrapper.

The future challenge of biomaterial‐based hydrogel film is to replace the function of thin plastic used as a wrapping. Plastic wrapping, as a single‐use plastic, is a significant source of plastic waste that needs to be reduced. Single‐use plastic consumption in the food and beverage sector significantly exacerbates the plastic waste problem, with projections showing an alarming increase in plastic production by 2050 if current practices continue [73]. The current policy direction of plastic use is toward zero waste and a circular economy. In terms of circularity, it is projected that the substitution of fossil‐based plastics will be gradual and could reach 25% by 2030 and 65% by 2050 [74]. Wrapping is expected to protect the product and regulate the product’s respiration rate, thereby extending the shelf life of fresh products. Hydrogel film as wrapping modifies the characteristics of biofilm, which is not just a thin layer like plastic, but is expected to improve its function as primary packaging. MCC/PVA/CMC‐based biofilms were developed from the banana pseudo‐stem [75], chitosan‐based biofilm [76], CMC composite biopolymer film/mycogenic selenium nanoparticles [77], CMC, and gellan gum with honokiol‐β‐cyclodextrin inclusion complex [78], and CMC‐based biofilm incorporating zeolitic imidazolate [79].

The hydrogel film (Figure 10(a)) was composed of CMC and MCC biomaterials derived from OPEFB waste, using a citric acid‐based green crosslinker. Applications in the food system must be able to prepare packaging that is not only environmentally friendly but also safe for products and consumers. Therefore, this hydrogel film can be further developed to replace polyethylene cling wrap for wrapping (Figures 10(b) and 10(c)) and increase the substitution of synthetic plastic materials. Therefore, the use of biomaterial films equivalent to polyethylene cling wrap in food products can be further increased. This is considering that about 36% of all plastic produced is used to make packaging, 85% of which ends up in landfills [80]. The development of CMC/MCC‐based hydrogel film from OPEFB promises to substitute the need for disposable wrapping packaging, with its advantage of being biodegradable and compatible with food products.

FIGURE 10Application of hydrogel film, (a) hydrogel film from CMC/MCC/citric acid, (b) dry wrapping methods, and (c) wet wrapping methods.

**Figure:**
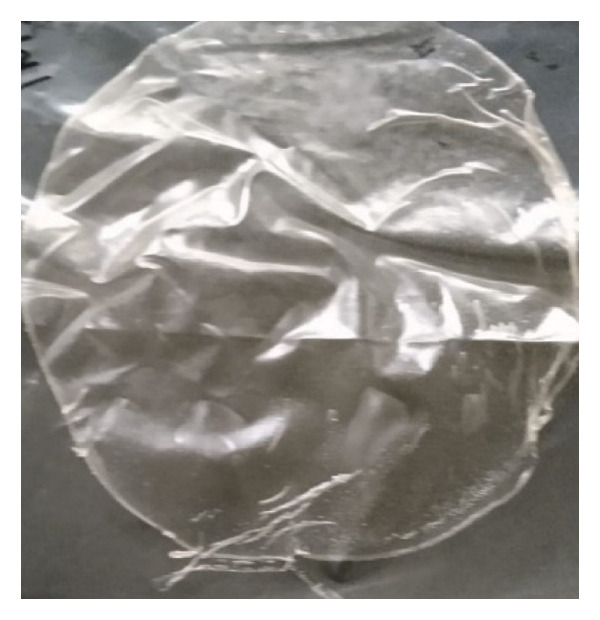
(a)

**Figure:**
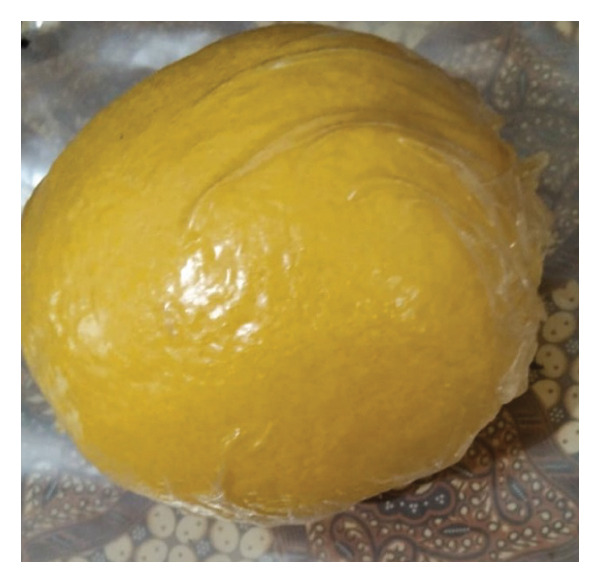
(b)

**Figure:**
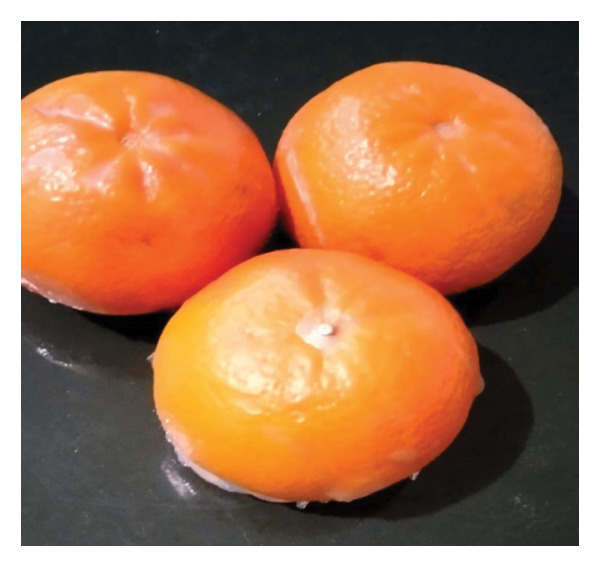
(c)

## 4. Conclusion

CMC‐ and MCC‐based hydrogel films were prepared using cellulose sourced from OPEFB, a byproduct of the palm oil sector. This hydrogel film is designed as one of the biomaterials to substitute for disposable PE cling wrap. CMC/MCC 90:10 formulation at 5% citric acid showed a good water absorption rate, mechanical strength, and no shedding. At the same concentration of citric acid, the CMC/MCC 80:20 formulation exhibited a 600‐fold higher water absorption quality but eventually decayed. This is confirmed by the sharpening of the wave number at 1705 cm^−1^, which is sharper in CMC/MCC 80:20 and correlates with the presence of carbonyl groups, as well as improved water absorption properties. XRD results will correlate with crystallinity, where higher crystallinity corresponds to lower water absorption properties due to increased density resulting from crosslinks. This is linear, with the surface properties of the CMC/MCC 90:10 being smoother. In contrast, the surface strains formed in CMC/MCC are visible as traces of water absorption in the dried hydrogel film. The ability to maintain structure in CMC/MCC 90:10 5% citric acid correlates with better thermal stability. Biomaterials in CMC/MCC hydrogel films, utilizing citric acid as a green crosslinker, still have limited information on their use. This hydrogel film has the potential to be developed as a wrapping material that needs to balance hydrophobicity, hydrophilicity, and plasticity.

## Author Contributions

Susi Susi and Makhmudun Ainuri conceived and administered the study. Methodology and formal analysis were developed by Susi Susi, Makhmudun Ainuri, Wagiman Wagiman, Mohammad Affan Fajar Falah, and Hisyam Musthafa Al Hakim. Investigation, validation, and data analysis were conducted by Susi Susi, Makhmudun Ainuri, and Hisyam Musthafa Al Hakim, with Susi Susi leading the software development and Makhmudun Ainuri handling the graphic design. Susi Susi drafted the manuscript, which was reviewed by all authors. Makhmudun Ainuri, Wagiman Wagiman, and Mohammad Affan Fajar Falah conducted the experiments.

## Funding

This study was supported by Indonesia Oil Palm Plantation Fund Management Agency (BPDPKS), PRJ‐70/DPKS/2023.

## Disclosure

All authors approved the final version.

## Conflicts of Interest

The authors declare no conflicts of interest.

## Data Availability

Data available are upon request from the authors.
